# An information model for computable cancer phenotypes

**DOI:** 10.1186/s12911-016-0358-4

**Published:** 2016-09-15

**Authors:** Harry Hochheiser, Melissa Castine, David Harris, Guergana Savova, Rebecca S. Jacobson

**Affiliations:** 1 Department of Biomedical Informatics, University of Pittsburgh School of Medicine, 5607 Baum Boulevard, Rm 523, Pittsburgh, 15206-3701 PA USA; 2 Intelligent Systems Program, University of Pittsburgh, Pittsburgh, PA USA; 3 Boston Children’s Hospital and Harvard Medical School, Boston, MA USA; 4 University of Pittsburgh Cancer Institute, Pittsburgh, PA USA

**Keywords:** Cancer, Deep phenotyping, Information extraction, Information model

## Abstract

**Background:**

Standards, methods, and tools supporting the integration of clinical data and genomic information are an area of significant need and rapid growth in biomedical informatics. Integration of cancer clinical data and cancer genomic information poses unique challenges, because of the high volume and complexity of clinical data, as well as the heterogeneity and instability of cancer genome data when compared with germline data. Current information models of clinical and genomic data are not sufficiently expressive to represent individual observations and to aggregate those observations into longitudinal summaries over the course of cancer care. These models are acutely needed to support the development of systems and tools for generating the so called clinical “deep phenotype” of individual cancer patients, a process which remains almost entirely manual in cancer research and precision medicine.

**Methods:**

Reviews of existing ontologies and interviews with cancer researchers were used to inform iterative development of a cancer phenotype information model. We translated a subset of the Fast Healthcare Interoperability Resources (FHIR) models into the OWL 2 Description Logic (DL) representation, and added extensions as needed for modeling cancer phenotypes with terms derived from the NCI Thesaurus. Models were validated with domain experts and evaluated against competency questions.

**Results:**

The DeepPhe Information model represents cancer phenotype data at increasing levels of abstraction from mention level in clinical documents to summaries of key events and findings. We describe the model using breast cancer as an example, depicting methods to represent phenotypic features of cancers, tumors, treatment regimens, and specific biologic behaviors that span the entire course of a patient’s disease.

**Conclusions:**

We present a multi-scale information model for representing individual document mentions, document level classifications, episodes along a disease course, and phenotype summarization, linking individual observations to high-level summaries in support of subsequent integration and analysis.

## Background

Our ability to deeply investigate the cancer genome is outpacing our ability to correlate genetic changes with the phenotypes that they produce. Advances in tumor genomic profiling allow for the possibility of detailed molecular classification of cancers, potentially including whole exome or whole genome sequences derived from multiple tumor locations and peripheral blood, collected at multiple time points during tumor progression. However, methods and tools for linking these rich genomic data to relevant clinical information remain quite limited. Many key phenotypic variables in cancer including tumor morphology (e.g. histopathologic features), laboratory findings (e.g. gene amplification status), specific tumor behaviors (e.g. metastasis), and response to treatment (e.g. effect of a chemotherapeutic agent on tumor volume) are available only in clinical notes, or are fragmented across multiple data sources.

To better serve translational researchers, new techniques are needed to extract and represent these phenotypes from electronic health record (EHR) data. The set of features representing the clinical expression of the disease over time can be defined as the *deep phenotype* - “the precise and comprehensive analysis of phenotypic abnormalities in which the individual components of the phenotype are observed and described for the purposes of scientific examination of human disease” [1]. Cancer deep phenotypes will integrate data from both structured and unstructured clinical records, as well as patient reported measures, to form longitudinal models of each patient’s course.

The long-term goal of our research is to develop a generalizable computational infrastructure that will facilitate the extraction, manipulation, and use of these deep phenotypes, combining them with genomic data to drive discovery and precision medicine. As a first step towards this goal, we present an information model for cancer phenotypes, derived from translational cancer research programs and validated by cancer researchers working in three domains: breast cancer, ovarian cancer, and melanoma. We extend and complement evolving FHIR models [2], defining cancer-specific extensions for describing tumors, treatments, metastases, recurrences, and other key factors, at levels of abstraction varying from specific mentions in clinical notes to distinct episodes of care (e.g. staging, treatment, and follow-up) to summative descriptions of patients.

The information model will play a key role in a National Cancer Institute (NCI)-funded collaboration to develop new methods for extracting, representing, and visualizing cancer deep phenotypes. In the future, we expect to use these models to provide the foundation for expressive and interactive phenotype exploration tools [3] supporting cohort identification and analysis for cancer research.

### Cancer deep phenotype extraction

Extraction and representation of cancer phenotypes is typically a manual curation process. For specific cancer diagnoses, hospital and state cancer registries provide retrospective manual abstraction of clinical observations including outcomes and some phenotypic attributes. However, cancer registries often lack treatment and recurrence information critical for addressing retrospective research questions [4]. Consequently, a major effort of many NCI designated Cancer Centers, NCI Specialized Programs of Research Excellence (SPOREs), and Cancer Cooperative Groups has been to obtain detailed, structured phenotypic data [5, 6]. The collection of TCGA clinical data is a well-known example of a cancer data requiring manual abstraction for phenotype representation [7]. The TCGA dataset includes data from more than 100 institutions contributing structured phenotype data along with biomaterials for high throughput molecular classification on over ten thousand cancer cases.

Previous work in a number of NIH-funded translational science initiatives, such as eMERGE, has demonstrated the benefits of natural language processing (NLP) methods for cohort identification in both genome-wide [8–10] and phenome-wide [11] association studies. However, these initiatives have focused almost exclusively on non-cancer phenotypes, and have had the goal of dichotomizing patients for a particular phenotype of interest (for example, Type II Diabetes). Less focus has been given to identifying specific key variables such as response to treatment and extent of disease, or to extracting and representing the temporal aspects of disease progression and treatment.

Our ongoing work on natural language processing (NLP) systems provides important experience relevant to computable cancer phenotypes. The TIES project applies NLP techniques to the extraction of cancer phenotype data from clinical notes [12, 13], but the resulting models lack necessary granular phenotype detail and summarization over time. The cTAKES system has also been used for annotation of a variety of cancer specific variables and has the advantage of annotating temporal expressions and relations [14–20], but similarly focuses on the extraction of mentions within documents, lacking a phenotype level representation.

### DeepPhe cancer information model

Our goal was to build a cancer information model to provide a series of progressively more abstract representations suitable for aggregating individual observations from clinical text or structured data into summarizations of *Documents*, *Episodes*, and eventually individual patient *Phenotypes* [21]. For example, multiple mentions of a chemo-therapeutic agent in a single clinical note (and corresponding medication administration record) might be combined to form a *Document* summary indicating the specific drugs and dosages. Several documents with similar records occurring over several weeks might be further summarized as a treatment *Episode*, with still further summarization listing the set of agents as a *Treatment Regimen*, associated with adjuvant therapy for the primary tumor, and producing radiographic evidence of response as part of the Tumor *Phenotype. Mention*, *Document* and *Episode* represent fundamentally different levels of abstraction, all of which must be considered to accurately assess the *Phenotype*, when inferring from clinical data.

The DeepPhe information model includes these multiple levels of representation, along with provenance information linking the higher-level abstractions to their underlying individual statements [22], as necessary for verifying the accuracy of summarized information. Our initial implementation operates on entities extracted via the cTAKES NLP system, providing both a functional implementation and a demonstration of how this approach might be adapted to work with other NLP tools.

Because the sequence of events can influence the resulting phenotype, information models must provide informative representations of the temporal relationships between events. Previous efforts have proposed temporal models for clinical events [18, 23–26]. Recent projects, including SemEval clinical TempEval [27] and THYME provide insights into the automated annotation of temporal events, expression and relations [14, 28], which can support more sophisticated temporal reasoning. Ideally, temporal cancer phenotype models will facilitate the aggregation of such detail from individual healthcare encounters into abstractions corresponding to key epochs in cancer care such as diagnosis, surgery, treatment, and progression [29].

In the spirit of community efforts such as the OBO-Foundry [30], information models should build upon existing community standards and models wherever possible. Relevant efforts specific to cancer include the NCI Metathesaurus and NCI Thesaurus [31] as well as the Cancer Data Standards Repository (caDSR) [32]. Ontological efforts such as the Human-Phenotype ontology [33] and the Disease Ontology [34] provide well-organized terms and relationships for individual phenotypes, however, they do not provide the structure necessary for creating detailed descriptions of individual patients. Clinical element models (CEMS) [35–38] and the emerging Fast Healthcare Interoperability Resources (FHIR) [39–41] provide the necessary structure for representations but have thus far been focused on low-level elements and have not been used to develop summarizing abstractions for phenotypes. In this manuscript, we present a cancer deep phenotype information model that builds on underlying standards and terminologies to meet these and related requirements (Table 1).

**Table 1:** Modeling requirements

	Requirement	Description
R1	Appropriate terminology	Use accepted terminologies and vocabularies whenever possible
R2	Cancer-specific content	Provide expressivity necessary to develop appropriately detailed descriptions of cancer treatment and progression
R3	Available tooling	Align with existing APIs, schemata, validators, etc.
R4	Community-driven modeling	Use community contributions and critiques to improve models
R5	Compatibility with existing NLP infrastructure	Facilitate interaction with existing NLP tools and type systems.
R6	Combinations of structured and unstructured data	Support the combination of structured data represented in EMRs with unstructured details extracted from clinical texts.
R7	Multi-level modeling	Support summarization of data across multiple levels of abstraction, ranging from instances/mentions to documents, episodes (collections of records indicating a distinct phase in disease progression such as diagnosis or treatment), and high-level summaries of cancers and tumors.
R8	Provenance	Preserve and expose linkages between abstracted models and source data

## Methods

**Fig. 1:**
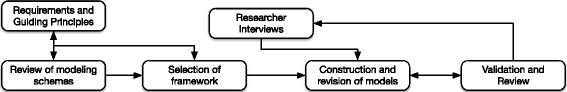
A schematic representation of the workflow used by the authors to generate the FHIR cancer models

The development of the cancer phenotype models involved sequential steps consistent with recently published process models for clinical information model development [42], including (1) review of prior schemata, (2) development of guiding requirements (Table 1), (3) interviews with domain experts, (4) selection of an appropriate standard and/or formal method framework, and (5) iterative model development, validation, and review (Fig. 1). We also used descriptions of user personae to inform model development. Additional methodological details can be found at https://github.com/deepphe/models/wiki.

### Selection of modeling framework

We reviewed existing models of clinical and biomedical data to identify formalisms for representing cancer deep phenotypes, modeling languages with appropriately expressive semantics, and vocabularies sufficient for communicating details of cancer diagnosis and treatment. We used this review to develop a list of requirements for our information model, including use of appropriate terminology providing required coverage of cancer concepts (Requirement R1); flexibility and extensibility (R2); availability of tooling including validators and application programming interfaces (APIs) (R3); the possibility of using community input to drive the development and evolution of our models (R4); easy integration with existing NLP tools (R5); the need to support both structured and unstructured data (R6); modeling at multiple levels of granularity (ranging from text spans in documents to patient-level summaries) (R7); and inclusion of provenance linkages between individual data items and higher-level summaries (R8). We evaluated four possible formalisms against these requirements, including clinical element models [35–38], caDSR information models [32, 43], OBO-Foundry biomedical ontology models [30] including the entity + quality framework [44], and FHIR [39–41].

FHIR offers significant strengths which include detailed schemata suitable for validation, reference implementations (R3), an extensive collection of software designs and tools, including proposed extensions to handle the inclusion of genomic information in EMRs [39], and an active community of developers (R4). The FHIR XML definitions are also easily convertible to a format compatible with the type system used by the cTAKES NLP Suite [45] (R5), which we plan to use to extract deep phenotypes from clinical notes. FHIR’s models of observations, diagnostic reports and medications are well suited for representing available structured information (R6). For these reasons, we selected FHIR as the underlying modeling formalism for our cancer phenotype models. Our model development included the extension of FHIR resources to model cancer concepts such as tumors (R1, R2), as necessary for the required multi-level representation of cancer phenotypes (R7). Provenance relations between levels in the model enumerate the linkages between lower-level details and more abstract summaries (R8).

We considered the NCI-Thesaurus [31] and OBO-Foundry [30] ontologies as candidate cancer vocabularies. Although there is some coverage of cancer-related phenotypes, both in broader ontologies such as the disease ontology [34] and the human-phenotype ontology [33] and in some domain-specific cancer ontologies [46, 47], we were not able to identify OBO ontologies that provided the detailed phenotype entities and attributes needed to represent the subtleties inherent in cancer progression and treatment. The NCI Thesaurus [31] was therefore chosen as the richest available set of curated cancer terms and concepts.

Our models are based on a translation of FHIR structure to an OWL 2 Description Logic (DL) representation [48]. OWL offers several advantages aligned with the goals of the DeepPhe project, including a semantic infrastructure suitable for representing both structured and unstructured data (R6); constraints appropriate for many of the domain-specific requirements of cancer modeling (R1, R2); the availability of reasoners and rule systems needed for managing summarization (R3); and the potential for compatibility with community ontology processes (R4, R5), especially those linking phenotypic and genomic information [25, 37, 49]. OWL also provides for the possibility of incorporating data provenance references (R6) [22].

Our next steps include expanding our model to include additional details necessary for the representation of cancer phenotypes for ovarian cancer, and malignant melanoma using data from interviews already collected, and then to add additional models for other solid tumors using the same basic methods. We also plan to align our OWL representations with ongoing community efforts to develop a FHIR representation. Although these efforts began in the fall of 2014 [50], community proposals were not complete at the time of this writing. We will align with HL7/W3C models for FHIR in RDF as they progress toward community consensus.

### Construction of draft models

Development of initial draft models was based on an exploration of existing models from prior efforts, and discussion with collaborators. Cancer specific attributes and corresponding terminologies and value sets were developed based on existing data models provided to us by multiple groups of collaborating cancer researchers. In a parallel process, we reviewed the emerging FHIR model definitions [2] to identify resources appropriate for modeling basic clinical content (medications, procedures, observations, etc.). As compatibility with existing NLP systems was a key goal, we also examined FHIR models in the context of existing elements in the cTAKES model [45, 51], developing prelimimary mappings sufficient for using cTAKES to populate FHIR models. The cTAKES model is based on the SHARP secondary use Clinical Element Models [38]. Published models of care trajectories [29] informed the development of models for episodes. Abstract classes summarizing phenotypes, tumors, and cancers were developed through graphical concept maps and refined through a series of design discussions.

Input from domain experts informed the selection of candidate models and model attributes including information related to diagnosis, staging, biomarker status, adequacy of surgical resection, therapy, outcome, and other cancer-specific factors. Initial drafts were produced by manual merging and mapping of multiple information models, data dictionaries, and spreadsheets obtained from each domain group. Outcomes of this process were collected in a spreadsheet grouped by content area (e.g. demographics, clinical exam, family history, pathology, radiology, treatment, clinical genomic features, outcome), in which each row represented a single data element, along with a definition (where available) and example values. Each data element (each row) in the draft model was then transferred to an individual index card along with example values, to be used in the information modeling interviews (described below). To provide modularity and encourage reuse, general concepts were modeled in a shared file (cancer.owl) augmented with specialized extensions for breast cancer (breastCancer.owl) with further extensions under development for other cancers.

OWL definitions were created through a manual translation process. XML FHIR definitions for a subset of the FHIR Resource models (e.g. Condition, Procedure, MedicationStatement, Observation, BodySite, Patient and CarePlan) and datatypes (e.g. Quantity, Range, Ratio, Period) were reviewed and translated into OWL using the Protégé ontology editor.

### Domain expert interviews

We conducted two different types of interviews to separately capture the process and content constraints for the models. For process, we conducted open-ended interviews using a modification of the Beyer and Holtzblatt Contextual Inquiry method [52]. For content, we conducted information modeling interviews that included card sorts of potential data elements. Information modeling interviews were conducted with funded collaborators; contextual inquiry interviews were classified as exempt by the University of Pittsburgh Human Research Protection Office (PRO13120154).

*Contextual Inquiry Interviews* with cancer researchers facilitated understanding of information needs, workflows, and practices to identify cohorts and related phenotypes to molecular characteristics. In interviews conducted by author HH, all participants were asked to describe their research goals and questions, and to either directly illustrate (when possible) or describe their use of informatics tools to meet those goals. Interviews were audio-coded and reviewed to extract descriptions of information needs, processes, and challenges [53]. Information needs identified through these discussions were used as input to the model development processes and to the development of competency questions; discussions of processes and challenges contributed to the development of work models that will inform the design of planned analytic tools (to be reported in a future publication).

*Information Modeling Interviews* with project collaborators involved in cancer research provided insights into the necessary content, relative importance and need for information extraction methods. For each of three cancer types (breast, ovarian, and melanoma), we separately interviewed one or more translational researchers actively engaged in using clinical data. For each domain, we also interviewed one or more data managers or abstractors, who were primarily responsible for obtaining clinical data from various sources including EMRs. In interviews conducted by author RJ, each participant was provided with the complete set of index cards representing all data elements in the draft model and asked to prioritize them on two axes.

For the first axis, they were asked to sort the cards based on whether they considered any given data element to be important information for (a) their own research, (b) for the research of colleagues, or (c) not important. They subsequently prioritized group (a) into those that were very important and somewhat important. For the second axis, they were asked to resort cards in group (a) and (b) based on whether they typically obtained such data from (a) structured electronic sources, (b) unstructured electronically available sources, or (c) unstructured sources not amenable to automated processing (e.g. paper charts, PDF documents).

Individuals completed either both card sorts or only one card sort based on their roles on the research team. We also asked each participant to add data elements that were important to the research team, but were not represented in the card set, and to include these additional data elements in both sorts. Throughout the interview process, the interviewer and participant engaged in an ongoing refinement of the meaning and importance of various data elements. Cards were marked during the interviews to capture prioritization on both axes. Interviews were captured on audio recorder, and transcribed verbatim. Transcriptions and prioritizations were analyzed to further identify, refine and categorize data elements, value domains, valid values (for enumerated elements), priority, and availability. The results were used to guide revision of the models.

### Model revision

Initial models were refined through an iterative process involving both domain expert feedback and review against sample clinical notes. Data elements, relationships, and value sets were revised based on feedback from the card sorting activities conducted during the information modeling interviews. Values and value domains provided by researchers were included. We manually populated instances of candidate models using a sample of de-identified clinical notes from cancer patients and compared those models to the original notes to verify sufficient expressivity. This review identified both new data elements and new linguistic modifiers describing negation, hedging, temporality, and other qualifiers for inclusion in the value sets. These items were added to the model and the process was repeated with an additional set of documents.

### Model validation

Finally, candidate models generated from this sequence of activities were presented to domain experts to validate that the information of interest in a set of reports could be represented accurately. The process was conducted as a presentation by the original information modeling interviewer. For each major part of the domain model, we presented the expert with example text, highlighted to show entities and their relationships in tandem with the associated representation. For example, we depicted a cancer *Phenotype* as the sum of information deriving from the *Primary Tumor* as well as the subsequent *Metastatic Tumor*. For each major modeling decision, we also asked experts to comment on the appropriateness of this method for the specific example. For example, we defined an initial set of *Episode* types corresponding to important intervals in a patient’s *Disease Course* and had the experts review them to confirm that they were correct. Results informed the final candidate models which were then prepared for release.

## Results

### Informant interviews

A total of 13 interviews were performed with domain experts, including 6 contextual interviews and 7 information modeling interviews. Interviews were performed with translational researchers and their staff working in the areas of breast cancer, ovarian cancer, and melanoma between October 2014 and August 2015. Participants included principal investigators, research fellows, and clinical data managers. Interview lengths ranged from approximately 1 to 2 h.

For information modeling interviews, the total number of data elements considered in the card sort was 137 (breast cancer), 86 (ovarian cancer) and 97 (melanoma). Of the total, 16 (breast cancer), 15 (ovarian cancer) and 25 (melanoma) new data elements were added by the participants.

#### Prioritization

For the breast cancer model, informants prioritized 101/137 data elements as specifically important to them, 35/137 data elements as potentially important only to other researchers but not to themselves, and 1/137 data elements as not important to themselves or other researchers. For the ovarian cancer model, informants prioritized 81/86 data elements as specifically important to them, 4/86 data elements as potentially important to other researchers but not to themselves, and 1/86 data elements as not important to themselves or to other researchers. For the melanoma model, informants prioritized 86/97 data elements as specifically important to them, 9/97 data elements as potentially important to other researchers but not to themselves, and 2/97 data elements as not important to themselves or to other researchers.

#### Availability

Of the total data elements, research staff currently tasked with collecting this data indicated that the large majority of these data elements could only be manually abstracted at present. This included 112/137 data elements for breast cancer, 79/86 data elements for ovarian cancer and 90/97 data elements for melanoma which are currently and routinely abstracted from free text electronic medical records. Structured data is available for only a small number of data elements in each model.

#### Overlap among individual models

Models for breast cancer, ovarian cancer and melanoma contained significant overlap with 52 data elements shared by all three models. These included key variables such as tumor stage, treatment, and outcome. In contrast, 129 data elements were unique to a specific domain. These included specific types of somatic mutations (e.g. BRAF), germline mutations (e.g. BRCA1), biomarkers (e.g. CA125), risk factors (e.g. UV exposure) prognostic features of the tumor (e.g. tumor infiltrating lymphocytes), and specific clinical features (e.g. associated ascites).

### Information model

**Fig. 2:**
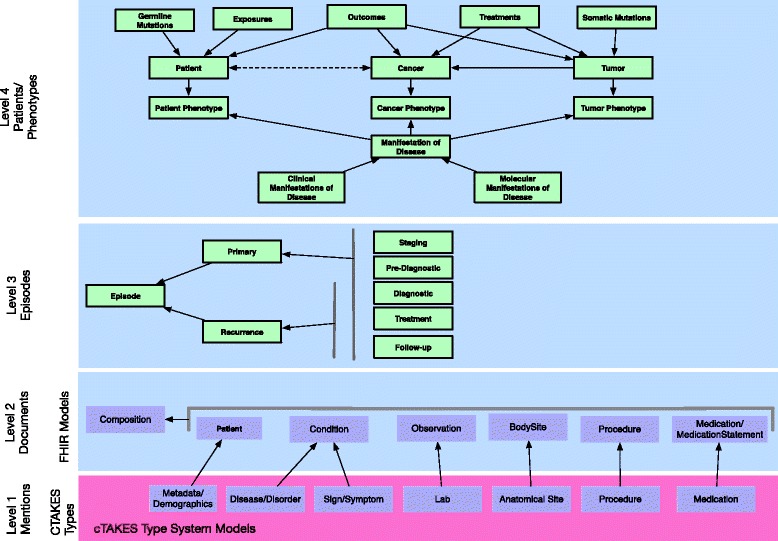
Classes used in cancer phenotype representations. Individual mentions extracted from NLP (Level 1) are instantiated as FHIR Objects, which are collected in Compositions corresponding to individual documents (Level 2). These FHIR objects become events that are aggregated into distinct Episodes of care (Level 3) and eventually analyzed to form patient and phenotype level summaries (Level 4)

Figure 2 provides an overview of the information model including the four constituent levels: Mention, Document, Episode, and Phenotype.

#### Mentions (Level 1)

*Mentions* are represented using the cTAKES type system, which provides an interoperability standard based on the SHARP secondary use Clinical Element Models. This data provides essential building blocks for higher-level summarization of *Documents, Episodes and Phenotypes*. For temporal representation, we reuse entities articulated in the cTAKES temporal module, including events, document time relations (DocTimeRel) that build on the classic Allen temporal relations [54], and the notion of temporal containers.

#### Composition (Level 2)

**Fig. 3:**
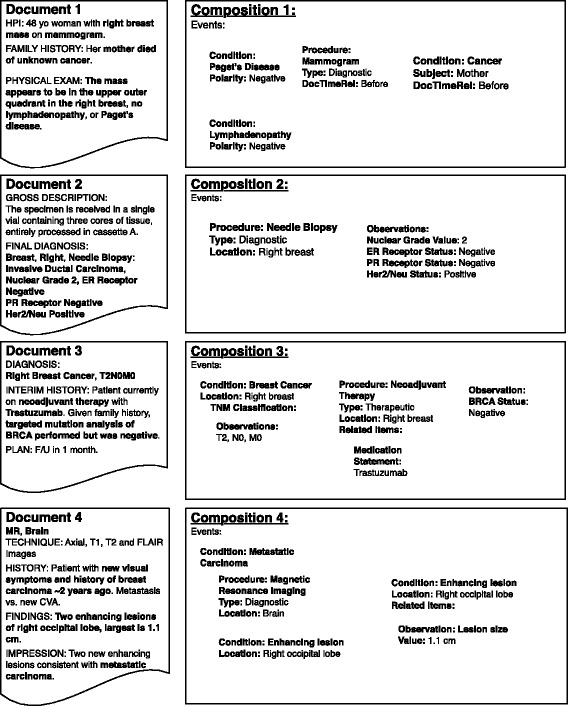
Example patient records and their representation as compositions

Individual mentions and their relations are combined into a composition model representing all details from an individual clinical note. As an example, Document 1 in Fig. 3 includes multiple mentions of a *Mass* that is summarized into one event detail in the corresponding composition model. Data captured in Level 1 cTAKES types can be transformed to Level 2 FHIR resources which are aggregated to create FHIR compositions (R5), and stored as event details (R7). Resources selected as an initial subset of the FHIR Data Standard for Trial Use 2 include *Condition*, *Patient*, *Observation, BodySite, Procedure,* and *MedicationStatement*, which were sufficient for modeling a large number of concepts extracted from clinical text via NLP. OWL classes from an existing NLP information extraction schema [55] were modified to represent this subset of FHIR resources.

#### Episodes (Level 3)

**Fig. 4:**
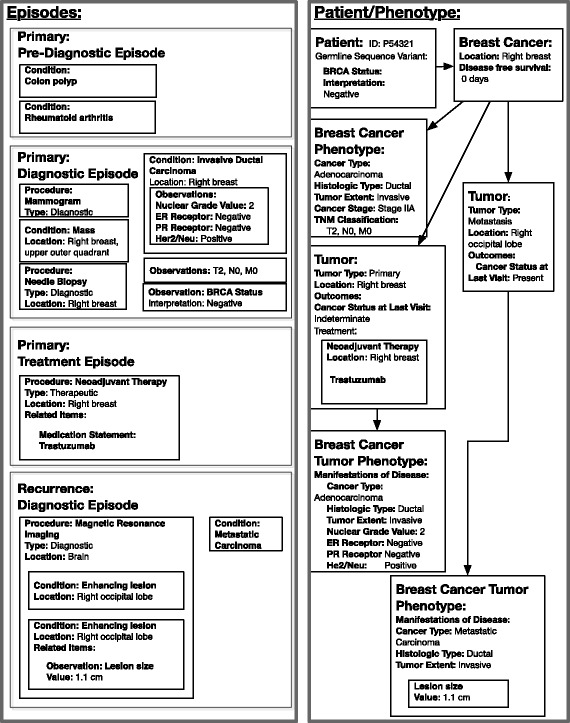
Summarization of records from Fig. 3 into Episodes and Patient/Phenotype Summary

At the Episode level, we model specific disease-relevant intervals with expected key events, as *Episodes* within a *Disease Course*, extending previous work on cancer trajectories [29]. *Events* are contained and ordered within these disease-relevant episodes. Episodes are hierarchical (containing other episodes), may overlap, and include start and end dates as well as start and end events. For example, a *Primary Tumor* episode is composed of constituent episodes (phases) including a *Diagnostic* episode. The diagnostic episode begins with the presentation of a complaint, symptom, or sign that initiates a diagnostic workup, and ends with a pathologic, laboratory, or radiologic diagnosis of a new *Cancer*. Episodes are defined as extensions of the FHIR Bundle class and can be ordered to form an abstracted timeline of a patient’s *Disease Course* (Fig. 4). Thus, events from Documents 1, 2 and 3 in Fig. 3 (e.g. mammogram, mass, needle biopsy, invasive ductal carcinoma, T1, N0, M0, BRCA status) are classified as belonging to a *Primary Tumor* episode in the *Diagnostic* phase whereas the events from Document 4 (e.g. MRI, enhancing lesion, metastatic carcinoma) are classified as belonging to a *Metastatic Tumor* episode in the *Diagnostic* phase. Extraction of subsequent entities, events, and relations can thus be conditioned on the unique context of the disease-specific episode. Visualization and search methods can also leverage the context to return more relevant results.

#### Phenotypes (Level 4)

At the phenotype level, we model abstractions of key variables over time. Disease indicators are grouped under *Manifestations of Disease*, a class subsuming *Clinical Manifestations* (e.g. cancer type, stage, histology, etc.) and *Molecular Manifestations* (e.g. receptor status). *Cancer*, *Tumor*, *Cancer Phenotype*, and *Tumor Phenotype* classes represent the overall progression and treatment of cancers and individual solid tumors, with *Cancer* summarizing details relavent to the disease as a whole, and *Tumor* summarizing details relevant to individual mass-occupying lesions. *Cancers* may exhibit different *Cancer Phenotypes* over time, just as *Tumors* may exhibit more than one *Tumor Phenotype. Cancers* and *Tumors* may be treated, thus altering the phenotypes with which they are associated. For example, a given tumor may have the phenotype “ER positive” at one point in time, but may acquire the phenotype “ER negative” at another point in time. Example attributes of *Cancer Phenotype* and *Tumor Phenotype* are shown in Table 2. *Phenotypes* also include co-morbidities, including those that are relevant to cancer. Additional classes describe *Treatments, Outcomes*, and *Germline Sequence Variations* and *Tumor Sequence Variations*. Wherever possible, phenotype level entities are defined within existing biomedical ontologies, favoring those developed using OBO principles. For example, *Germline Sequence Variation* links to the Sequence Ontology class, *sequence_variant*, by referencing the class id (SO:0001060) in the rdfs: seeAlso annotation property.

**Table 2:** Attributes of (a) cancer and (b) Tumor phenotypes

(a)	
Cancer Phenotype	
Cancer Type	carcinoma, sarcoma, etc.
Histologic Type	ductal, lobular, etc.
Tumor Extent	in-situ, invasive, etc.
Cancer Stage	Stage I, Stage IIA, etc.
T Classification	Primary Tumor Classification (pTis, T2a, etc.)
N Classification	Regional lymph node classification (pNx, N1, etc.)
M Classification	Distant metastasis classification (M0, M1, etc.)
Manifestations	Clinical and Molecular classifications of the cancer (hypercalcemia, hypercoaguability, etc.)
(b)	
Tumor Phenotype	
Cancer Type	by cell of origin (carcinoma, sarcoma, etc.)
Histologic Type	ductal, lobular, etc.
Tumor Extent	in-situ, invasive, etc.
Manifestations	Clinical and Molecular classifications of the tumor (size, receptor status, Nottingham score, etc.)

Linkages between mentions, documents, and episodes are accomplished through provenance extensions to the FHIR resources (R8). Each higher-level resource refers to one or more lower-level resources, using the “prov:wasDerivedFrom” relationship from the PROV provenance ontology [22]. The transitive closure of these relationships, along with direct relationships between concepts such as *Cancer* and *Tumor*, will form a complete derivation path for the abstracted models.

The DeepPhe model is defined in publicly available OWL files [56] distributed under a creative commons Attribution International 4.0 license. Readers are encouraged to use GitHub code control and issue-tracking tools for the model repository to provide comments, suggest enhancements, and explore potential extensions and adaptations to the models (R4).

### Use of the model for phenotyping

**Fig. 5:**
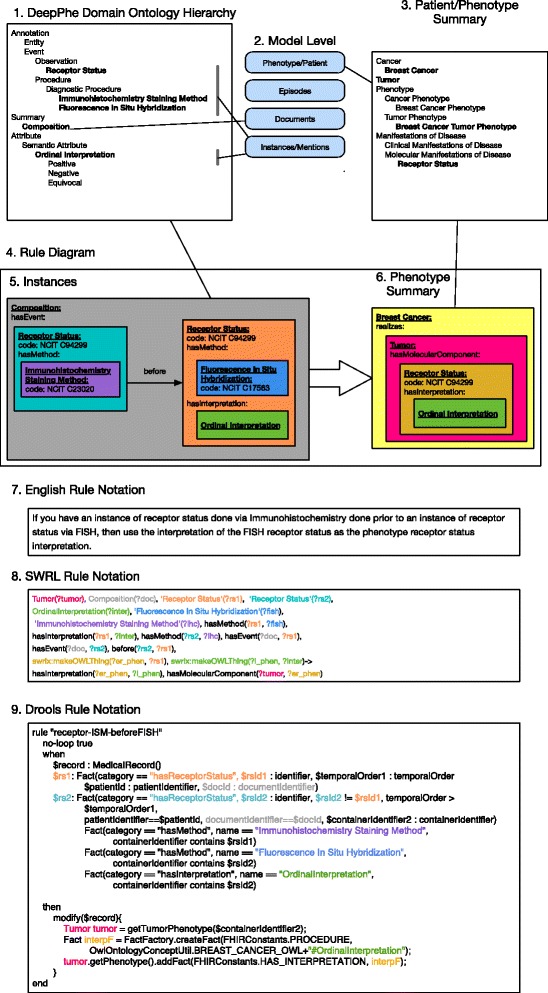
An example abstraction rule and its expression in SWRL. Summarization rules convert assertions extracted from individual documents into higher-level summaries. (1) A subset of the upper-levels of the information model showing key concepts in representation of both instance and summary models. (2) A mapping of those concepts to levels in the information model. (3) A subset of the elements used in a Patient/Phentoype level summary. (4) A graphical example of a rule taking instances (5) and transforming them into a summary representation (6). This rule indicates that the value of a FISH test will take precedence over results of an IHC test. This rule is given in English (7), SWRL (8), and Drools (9)

Construction of a patient phenotype is envisioned as a multi-step process. Currently we leverage the models described above (1) to produce dictionaries for a new concept recognition component of cTAKES [45] using the NobleCoder concept recognition tool [57], and (2) as the knowledge representation for developing phenotyping rules capable of combining individual observations from the mention level into appropriate instances of data elements at the phenotype level. Initial rules validating the approach were developed using the Semantic Web Rule Language [58], with subsequent rules implemented in the Drools system [59] (Fig. 5). In the future, we will extend our DeepPhe NLP pipeline to (3) leverage the model’s attributes and valid values to selectively process documents based on class (e.g. Breast Cancer, Ovarian Cancer) and (4) interdigitate EMR data, cancer registry data, and data derived from NLP pipelines as input to the phenotyping rules.

### Model validation

Both contextual inquiry and information modeling interviews (including card sorts) were used to develop a set of competency questions [60] suitable for validating the resulting cancer phenotype models. Competency questions reflect prototypical questions that might be asked by cancer investigators. Resulting questions encompass the identification of patients with specific clinical profiles, potentially including temporal relationships between sentinel events; comparison of patients by cohort; integration of information across multiple sources; and identification of available information. Sample competency questions are given in Table 3; full details are available at the project website [56].

**Table 3:** Sample competency questions

Category	Description	Sample question
Clinicalcriteria	Find patients matching some desired criteria, independent of temporal relations	Which patients have had atypical endometriosis?
Eventrel	Find patients who experience two or more clinically-relevant events, related by a specified time interval.	Which patients were given chemotherapy within eight weeks of their death?
Stratification	Given two sets of patients similar in key respects, compare certain outcomes based on stratification of categorical values such as care, phenotype, etc.	What portion of BRCA patients with PALB2 were given PARP inhibitor therapy?
Triangulation	Some information cannot be interpreted on the basis of any one source. Integration of related information from multiple sources is required to develop full understanding.	Which patients had medications that were ordered (as per physician charts), but not administered (as per Medication Administration Records (MARs) or nursing records)?
Schema	What information is available on which patients?	For which patients do I have a valid date of death?

## Discussion

Proponents of “deep phenotyping” argue for the importance of detailed phenotypic descriptions - generally in a computable form—as prerequisites for finely-grained analyses that stratify patients into previously unknown classifications, thus enabling more precise investigation and characterization of human disease [1, 61–64]. This is also a key goal of precision medicine, which will require much more sophisticated analysis of patient data to derive meaningful features for classification and prediction. Achieving both of these goals will require advances in extraction of key details from patient records and also in assembling those details into sufficiently expressive and flexible representations. Although previous efforts such as eMERGE have shown the potential of large-scale extraction of phenotypic information from both structured and unstructured data sources [9], the resulting classifications have typically been dichotomous, describing patients in terms of the presence or absence of one or more specified diseases. More detailed models are needed to build phenotype descriptions that capture the inherent complexity and diversity in the manifestations of human disease. Specifically, these models must convert individual facts and observations into computable phenotypes, describing patients at a level of granularity appropriate for interpretation of individual cases, comparison between cases, cohort selection, and hypothesis generation through exploration of large datasets.

The DeepPhe information model builds on entities that can be extracted from structured or unstructured data in medical records and aggregated into individual documents, episodes, and eventually into high-level phenotypic descriptions. Provenance linkages tying higher-level phenotypic representations to constituent observational details provide audit trails suitable for verifying the abstractions, while also enabling analyses to move between levels of abstraction as necessary for specific tasks.

The use of the HL7 FHIR data model bridges the gap between two key applications of patient data: data resulting from direct clinical care and secondary use of clinical data for research purposes. Although FHIR was clearly developed to meet clinical interoperability needs, the simple, well-documented designs of FHIR resources and data types, particularly including extension mechanisms, simplified the process of developing phenotype abstractions needed for translational research. This approach provides a model for adapting FHIR to support secondary use of clinical data, similar to efforts that use FHIR to integrate genomics into clinical records [39].

Our development of cancer-specific attributes and value sets raised familiar design issues such as pre- and post-coordination of descriptors [36] and differences in domain perspectives. As definitive answers to these questions are often not possible, our modeling efforts relied on a combination of pragmatism and reference to existing best practices. For example, biomarker test results (e.g. Estrogen Receptor, Progesterone Receptor and Her2Neu Receptor status) presented a challenge, as initial attempts to pre-compose testing methods, marker, and interpretation led to an unwieldy number of potential combinations. A post-composed model was chosen instead, with the understanding that equivalence classes would be added as needed.

Genetic and molecular descriptors present additional challenges for cancer modeling. We have included classes for both sequence variations and molecular manifestation resulting from those variants, with relationships between associated classes as necessary. User-facing tools based on these models might choose to combine these factors if necessary to align with the preferences of users in specific domains. We have chosen to adopt an initial model of structural variation that is significantly less detailed than the GeneticObservation suggested by the SMART on FHIR Genomics effort [39]. Subsequent evolution of our model will align more closely with this effort.

Our multi-level modeling approach identified—but did not fully resolve—modeling challenges associate with multiple uses of terms such as “tumor” or “cancer”. At the mention level, these terms refer to specific statements from clinical notes, while at the phenotype level they refer to abstractions of complex pathophysiologic events. Thus, we have included entities in our representation at both Level 1 and Level 4 that share the same names, although they refer to different conceptualizations. This duplication was deemed preferable to the creation of alternative terms.

Our cancer information model is presented here as a computable representation of longitudinal phenotype and treatment data at multiple levels of abstraction. Realizing the complexity and diversity of cancer treatment and disease progression, we do not expect that this approach is in any way final or definitive. We plan to engage an even broader range of informatics from the cancer research community to identify use cases suitable for extending the expressivity of the model and for guiding any necessary revisions. Extension of the models to handle non-solid cancers (e.g. leukemias) would be particularly useful for validation of the overall approach. Feedback can be provided at the project GitHub page, https://github.com/deepphe/models. We also plan to work with systems developers, cancer researchers engaged in complementary computational approaches such as tumor growth simulations [65, 66], and with related efforts such GA4GH [67], PhenoPackets [68], and others developing phenotype models [8] to identify additional use cases and opportunities for encouraging broader adoption and use of common methods and standards.

### Limitations

Although our modeling process combined both extensive interviews aimed at eliciting information needs from cancer researchers, review of relevant guidelines and standards for cancer care, and consideration of competency questions, our models are not expected to be generalizable to all use cases, both in terms of specific modeling decisions and scope of relevant concerns. We anticipate potential revisions to the model to accommodate the practicalities of working with functional NLP tools. Finally, FHIR RDF representations from the HL7/W3C community are evolving and (as of this writing) not fully complete. It is possible that completion of these efforts may lead to the adoption of some RDF models that are not directly compatible with our proposed information models. If any such inconsistencies arise, we will endeavor to ensure that subsequent revisions to our model are compatible with community-developed FHIR/RDF models to the greatest extent possible.

## Conclusion

Improved phenotypic descriptions of cancer and patient phenotypes are needed to advance translational research [69], quality care measures [4], and precision medicine [70]. We illustrate the potential benefits of using FHIR-compatible models, and offer a foundation suitable for extension to other domains.

We present a multi-level information model designed to support capture of cancer clinical data at multiple levels, from specific mentions in clinical texts, to summarization at increasingly higher levels of abstraction including documents, episodes, and phenotypes. The model is designed to be used by computational systems that extract these representations. Our model also provides an early example of rich representational models for deep phenotypes [69], suitable for adaptation, generalization, and community comment.

## Abbreviations

API: Application programming interface
BIRADS: Breast imagining reporting & data system
caDSR: Cancer data standards registry
CEM: Clinical element model
cTAKES: Clinical text analysis knowledge extraction system
DeepPhe: Deep phenotype
EMR: Electronic medical record
FHIR: Fast Health Interoperability Resources
NCI: National Cancer Institute
NIH: National Institutes of Health
NLP: Natural language processing
OBO: Open biomedical ontologies
OWL: Web ontology language
RDF: Resource description framework
SPORE: Specialized programs of research excellence
TCGA: The cancer genome atlas
TIES: Text information extraction system

## References

[1] Robinson PN (2012). Deep phenotyping for precision medicine. Hum Mutat.

[2] Index—FHIR v1.0.2 [http://hl7.org/fhir/]. Accessed 4 Sept 2016.

[3] Xu J, Rasmussen LV, Shaw PL, Jiang G, Kiefer RC, Mo H, Pacheco JA, Speltz P, Zhu Q, Denny JC, et al. Review and evaluation of electronic health records-driven phenotype algorithm authoring tools for clinical and translational research. J Am Med Inform Assoc. 2015;22(6):ocv070. 10.1093/jamia/ocv070 PMC5009915 26224336

[4] Hiatt RA, Tai CG, Blayney DW, Deapen D, Hogarth M, Kizer KW, Lipscomb J, Malin J, Phillips SK, Santa J et al. Leveraging state cancer registries to measure and improve the quality of cancer care: a potential strategy for California and beyond. J Natl Cancer Inst 2015, 107 (5):djv047 10.1093/jnci/djv047 25766400

[5] Helfand B, Roehl K, Cooper P, McGuire B, Fitzgerald L, Cancel-Tassin G, Cornu J-N, Bauer S, Van Blarigan E, Chen X et al. Associations of prostate cancer risk variants with disease aggressiveness: results of the NCI-SPORE Genetics Working Group analysis of 18,343 cases. Hum Genet. 2015;134(4):439–50. 10.1007/s00439-015-1534-9 PMC4586077 25715684

[6] Krumm R, Semjonow A, Tio J, Duhme H, Bürkle T, Haier J, Dugas M, Breil B. The need for harmonized structured documentation and chances of secondary use—Results of a systematic analysis with automated form comparison for prostate and breast cancer. J Biomed Inform. 2014;51:86–99. 10.1016/j.jbi.2014.04.008 24747879

[7] National Cancer Institute. TCGA Data Overview. https://wiki.nci.nih.gov/display/TCGA/TCGA+Data+Overview. Accessed 4 Sept 2016.

[8] Mo H, Thompson WK, Rasmussen LV, Pacheco JA, Jiang G, Kiefer R, Zhu Q, Xu J, Montague E, Carrell DS Xu J, Montague E, Carrell DS et al. Desiderata for computable representations of electronic health records-driven phenotype algorithms. J Am Med Inform Assoc. 2015;22(6):ocv112. 10.1093/jamia/ocv112 PMC4639716 26342218

[9] Newton KM, Peissig PL, Kho AN, Bielinski SJ, Berg RL, Choudhary V, Basford M, Chute CG, Kullo IJ, Li R et al. Validation of electronic medical record-based phenotyping algorithms: results and lessons learned from the eMERGE network. J Am Med Inform Assoc. 2013;20(e1):e147–54. 10.1136/amiajnl-2012-000896 PMC3715338 23531748

[10] Rea S, Pathak J, Savova G, Oniki TA, Westberg L, Beebe CE, Tao C, Parker CG, Haug PJ, Huff SM et al. Building a robust, scalable and standards-driven infrastructure for secondary use of EHR data: the SHARPn project. J Biomed Inform. 2012;45(4):763–71. 10.1016/j.jbi.2012.01.009 PMC4905766 22326800

[11] Denny JC, Ritchie MD, Basford MA, Pulley JM, Bastarache L, Brown-Gentry K, Wang D, Masys DR, Roden DM, Crawford DC. PheWAS: demonstrating the feasibility of a phenome-wide scan to discover gene–disease associations. Bioinformatics. 2010;26(9):1205–10. 10.1093/bioinformatics/btq126 PMC2859132 20335276

[12] Crowley RS, Castine M, Mitchell K, Chavan G, McSherry T, Feldman M: caTIES: a grid based system for coding and retrieval of surgical pathology reports and tissue specimens in support of translational research. J Am Med Inform Assoc 2010, 17 (3):253–264. 10.1136/jamia.2009.002295 PMC2995710 20442142

[13] Jacobson RS, Becich MJ, Bollag RJ, Chavan G, Corrigan J, Dhir R, Feldman MD, Gaudioso C, Legowski E, Maihle NJ et al. A federated network for translational cancer research using clinical data and biospecimens. Cancer Res. 2015;75(24):5194–201. 10.1158/0008-5472.CAN-15-1973 PMC4683415 26670560

[14] Lin C, Dligach D, Miller TA, Bethard S, Savova GK: Multilayered temporal modeling for the clinical domain. J Am Med Inform Assoc 2015, Oct 31. [Epub ahead of print]. 10.1093/jamia/ocv113 PMC5009920 26521301

[15] Lin C, Miller T, Kho A, Bethard S, Dligach D, Pradhan S, Savova G: Descending-Path Convolution Kernel for Syntactic Structures. In: Assocation for Compuational Linguistics Conference. Baltimore, MD 2014.

[16] Dligach D, Bethard S, Becker L, Miller T, Savova GK (2014). Discovering body site and severity modifiers in clinical texts. J Am Med Inform Assoc.

[17] Carrell DS, Halgrim S, Tran D-T, Buist DSM, Chubak J, Chapman WW, Savova G (2014). Using Natural Language Processing to Improve Efficiency of Manual Chart Abstraction in Research: The Case of Breast Cancer Recurrence. Am J Epidemiol.

[18] Albright D, Lanfranchi A, Fredriksen A, Styler WF, Warner C, Hwang JD, Choi JD, Dligach D, Nielsen RD, Martin J et. Towards comprehensive syntactic and semantic annotations of the clinical narrative. J Am Med Inform Assoc. 2013;20(5):922–30. 10.1136/amiajnl-2012-001317 PMC3756257 23355458

[19] Huang Z, Lu X, Duan H (2012). On mining clinical pathway patterns from medical behaviors. Artif Intell Med.

[20] Savova GK, Olson JE, Murphy SP, Cafourek VL, Couch FJ, Goetz MP, Ingle JN, Suman VJ, Chute CG, Weinshilboum RM. Automated discovery of drug treatment patterns for endocrine therapy of breast cancer within an electronic medical record. J Am Med Inform Assoc. 2012;19(e1):e83–9. 10.1136/amiajnl-2011-000295 PMC3392847 22140207

[21] Pivovarov R, Elhadad N (2015). Automated methods for the summarization of electronic health records. J Am Med Inform Assoc: JAMIA.

[22] Lebo T, Sahoo S, McGuinness D. "PROV-O: The PROV Ontology." 2013. https://www.w3.org/TR/prov-o/. Accessed 4 Sept 2016.

[23] Cherry C, Zhu X, Martin J, de Bruijn B (2013). À la Recherche du Temps Perdu: extracting temporal relations from medical text in the 2012 i2b2 NLP challenge. J Am Med Inform Assoc.

[24] Huff SM, Rocha RA, Bray BE, Warner HR, Haug PJ (1995). An event model of medical information representation. J Am Med Inform Assoc: JAMIA.

[25] Tao C, Solbrig HR, Chute CG: CNTRO 2.0: A harmonized semantic web ontology for temporal relation inferencing in clinical narratives. AMIA Joint Summits on Translational Science Proceedings AMIA Summit on Translational Science 2011, 2011:64–68. PMC3248753 22211182

[26] Tao C, Wei W-Q, Solbrig HR, Savova G, Chute CG: CNTRO: A Semantic Web Ontology for temporal relation inferencing in clinical narratives. AMIA Annu Symp Proc 2010, 2010:787–791. PMC3041418 21347086

[27] Bethard S, Derczynski L, Savova GK, Pustejovsky J, Verhagen M: SemEval-2015 Task 6: Clinical TempEval. . In: 9th International Workshop on Semantic Evaluation (SemEval 2015. Denver, Colorado; 2015.

[28] Sun W, Rumshisky A, Uzuner O (2015). Normalization of relative and incomplete temporal expressions in clinical narratives. J Am Med Inform Assoc: JAMIA.

[29] Defossez G, Rollet A, Dameron O, Ingrand P (2014). Temporal representation of care trajectories of cancer patients using data from a regional information system: an application in breast cancer. BMC Med Inform Decis Mak.

[30] Smith B, Ashburner M, Rosse C, Bard J, Bug W, Ceusters W, Goldberg LJ, Eilbeck K, Ireland A, Mungall CJ et al. The OBO Foundry: coordinated evolution of ontologies to support biomedical data integration. Nat Biotechnol. 2007;25(11):1251–5. 10.1038/nbt1346 PMC2814061 17989687

[31] Sioutos N, de Coronado S, Haber MW, Hartel FW, Shaiu W-L, Wright LW (2007). \NCI\ Thesaurus: A semantic model integrating cancer-related clinical and molecular information. J Biomed Inform.

[32] Komatsoulis GA, Warzel DB, Hartel FW, Shanbhag K, Chilukuri R, Fragoso G, de Coronado S, Reeves DM, Hadfield JB, Ludet C et al. caCORE version 3: Implementation of a model driven, service-oriented architecture for semantic interoperability. J Biomed Inform 2008, 41 (1):106–123. 10.1016/j.jbi.2007.03.009 PMC2254758 17512259

[33] Köhler S, Doelken SC, Mungall CJ, Bauer S, Firth HV, Bailleul-Forestier I, Black GCM, Brown DL, Brudno M, Campbell J et al. The Human Phenotype Ontology project: linking molecular biology and disease through phenotype data. Nucleic Acids Res. 2014;42(Database issue):D966–74. 10.1093/nar/gkt1026 PMC3965098 24217912

[34] Schriml L, Mitraka E. The Disease Ontology: fostering interoperability between biological and clinical human disease-related data. Mamm Genome. 2015;26:584. doi:10.1007/s00335-015-9576-9. 10.1007/s00335-015-9576-9 PMC4602048 26093607

[35] Lin K-W, Tharp M, Conway M, Hsieh A, Ross M, Kim J, Kim H-E. Feasibility of using Clinical Element Models (CEM) to standardize phenotype variables in the database of Genotypes and Phenotypes (dbGaP). PLoS One. 2013;8(9):e76384. 10.1371/journal.pone.0076384 PMC3776754 24058713

[36] Oniki TA, Coyle JF, Parker CG, Huff SM (2014). Lessons learned in detailed clinical modeling at Intermountain Healthcare. J Am Med Inform Assoc: JAMIA.

[37] Tao C, Jiang G, Oniki TA, Freimuth RR, Zhu Q, Sharma D, Pathak J, Huff SM, Chute CG. A semantic-web oriented representation of the clinical element model for secondary use of electronic health records data. J Am Med Inform Assoc : JAMIA. 2013;20(3):554–62. 10.1136/amiajnl-2012-001326 PMC3628064 23268487

[38] Wu ST, Kaggal VC, Dligach D, Masanz JJ, Chen P, Becker L, Chapman WW, Savova GK, Liu H, Chute CG. A common type system for clinical natural language processing. J Biomed Semantics. 2013;4(1):1. 10.1186/2041-1480-4-1 PMC3575354 23286462

[39] Alterovitz G, Warner J, Zhang P, Chen Y, Ullman-Cullere M, Kreda D, Kohane IS (2015). SMART on FHIR Genomics: Facilitating standardized clinico-genomic apps. J Am Med Inform Assoc.

[40] Jiang G, Solbrig HR, Kiefer R, Rasmussen LV, Mo H, Speltz P, Thompson WK, Denny JC, Chute CG, Pathak J. A standards-based semantic metadata repository to support EHR-driven phenotype authoring and execution. Stud Health Technol Inform. 2015;216:1098. PMC4898771 26262397

[41] Kasthurirathne SN, Mamlin B, Kumara H, Grieve G, Biondich P (2015). Enabling better interoperability for healthcare: lessons in developing a standards based application programing interface for electronic medical record systems. J Med Syst.

[42] Moreno-Conde A, Moner D, da Cruz WD, Santos MR, Maldonado M, Robles M, Kalra D (2015). Clinical information modeling processes for semantic interoperability of electronic health records: systematic review and inductive analysis. J Am Med Inform Assoc : JAMIA.

[43] Tobias J, Chilukuri R, Komatsoulis G, Mohanty S, Sioutos N, Warzel DB, Wright LW, Crowley RS (2006). CAP cancer protocols-a case study of caCORE based data standards implementation to integrate with the Cancer Biomedical Informatics Grid. BMC Med Inform Decis Mak.

[44] Gkoutos GV, Mungall C, Dolken S, Ashburner M, Lewis S, Hancock J, Schofield P, Kohler S, Robinson PN: Entity/quality-based logical definitions for the human skeletal phenome using PATO. Conf Proc IEEE Eng Med Biol Soc 2009, 2009:7069–7072. 10.1109/IEMBS.2009.5333362 PMC3398700 19964203

[45] Savova GK, Masanz JJ, Ogren PV, Zheng J, Sohn S, Kipper-Schuler KC, Chute CG. Mayo clinical Text Analysis and Knowledge Extraction System (cTAKES): architecture, component evaluation and applications. J Am Med Inform Assoc. 2010;17(5):507–13. 10.1136/jamia.2009.001560 PMC2995668 20819853

[46] Min H, Manion FJ, Goralczyk E, Wong Y-N, Ross E, Beck JR (2009). Integration of prostate cancer clinical data using an ontology. J Biomed Inform.

[47] Sojic A, Kutz O: Open biomedical pluralism: formalising knowledge about breast cancer phenotypes. Journal of Biomedical Semantics 2012, 3 Suppl 2 (Suppl 2):S3. 10.1186/2041-1480-3-S2-S3 PMC3448532 23046572

[48] Smith MK, Welty C, McGuinness DL. OWL Web Ontology Language Guide. 2004. https://www.w3.org/TR/owl-guide/. Accessed 4 Sept 2016.

[49] Tao C, Parker CG, Oniki TA, Pathak J, Huff SM, Chute CG. An OWL meta-ontology for representing the Clinical Element Model. AMIA Annu Symp Proc. 2011:1372–1381. PMC3243162 22195200

[50] RDF for Semantic Interoperability [http://wiki.hl7.org/index.php?title=RDF_for_Semantic_Interoperability]. Accessed 4 Sept 2016.

[51] Apache cTAKES [http://ctakes.apache.org/]. Accessed 4 Sept 2016.

[52] Beyer H, Holtzblatt K: Contextual Design: Defining Customer-Centered Systems San Francisco: Morgan Kaufman; 1998.

[53] Lazar J, Feng J, Hochheiser H (2009). Research Methods in Human-Computer Interaction.

[54] Allen JF (1983). Maintaining knowledge about temporal intervals. Commun ACM.

[55] Schema Ontology. [http://blulab.chpc.utah.edu/ontologies/v2/Schema.owl]. Accessed 7 Sept 2016.

[56] Cancer Deep Phenotype Extraction (DeepPhe) project information models [https://github.com/DeepPhe/models]. Accessed 4 Sept 2016.

[57] Tsetytlin E, Mitchell K, Legowski E, Corrigan J, Chavali G, Jacobson RS: NOBLE – Flexible concept recognition for large-scale biomedical natural language processing. BMC Bioinformatics, submitted. 10.1186/s12859-015-0871-y PMC4712516 26763894

[58] SWRL: A Semantic Web Rule Language Combining OWL and RuleML

[59] Drools, Drools - Drools - Business Rules Management System (Java™, Open Source). http://www.drools.org. Accessed 4 Sept 2016.

[60] Ren Y, Parvizi A, Mellish C, Pan J, Deemter KV, Stevens R. Towards Competency Question-driven Ontology Authoring. 11th ESWC 2014 (ESWC2014). 2014. http://data.semanticweb.org/conference/eswc/2014/paper/research/145. Accessed 4 Sept 2016.

[61] Bendall SC, Nolan GP (2012). From single cells to deep phenotypes in cancer. Nat Biotechnol.

[62] Frey LJ, Lenert L, Lopez-Campos G (2014). EHR Big Data deep phenotyping. Contribution of the IMIA Genomic Medicine Working Group. Yearb Med Inform.

[63] Kohane IS (2014). Deeper, longer phenotyping to accelerate the discovery of the genetic architectures of diseases. Genome Biol.

[64] Tracy RP (2008). ‘Deep phenotyping’: characterizing populations in the era of genomics and systems biology. Curr Opin Lipidol.

[65] Jeanquartier F, Jean-Quartier C, Schreck T, Cemernek D, Holzinger A: Integrating Open Data on Cancer in Support to Tumor Growth Analysis. In: Information Technology in Bio- and Medical Informatics: 7th International Conference, ITBAM 2016, Porto, Portugal, September 5–8, 2016, Proceedings. Edited by Renda EM, Bursa M, Holzinger A, Khuri S. Cham: Springer International Publishing; 2016: 49–66.

[66] Jeanquartier F, Jean-Quartier C, Cemernek D, Holzinger A (2016). In silico modeling for tumor growth visualization. BMC Syst Biol.

[67] Global Alliance for Genomica and Health. http://genomicsandhealth.org. Accessed 4 Sept 2016.

[68] PhenoPackets [http://phenopackets.org/]. Accessed 4 Sept 2016.

[69] Oellrich A, Collier N, Groza T, Rebholz-Schuhmann D, Shah N, Bodenreider O, Boland MR, Georgiev I, Liu H, Livingston K et al. The digital revolution in phenotyping. Brief Bioinform 2015. 10.1093/bib/bbv083 PMC5036847 26420780

[70] Precision Medicine Initiative (PMI) Working Group Report to the Advisory Committee to the Director, NIH. The Precision Medicine Initiative Cohort Program—Building a Research Foundation for 21st Century Medicine. 2015. http://acd.od.nih.gov/reports/DRAFT-PMI-WG-Report-9-11-2015-508.pdf. Accessed 4 Sept 2016.

